# From the Service of Prof. N. S. Davis, in the Medical Wards of Mercy Hospital, December 30, 1868

**Published:** 1869-03

**Authors:** W. A. Barstow


### FROM THE SERVICE OF PROF. N. S. DAVIS, IN THE MEDICAL WARDS OF MERCY HOSPITAL, December 30, 1868.

Reported by W. A. BARSTOW.

Gentlemen:—This young man came here from Waukegan, for the purpose of a careful examination of his case. He states that about one year ago he received an injury, by being struck by a wheel weighing about 50 lbs., which was thrown off the shaft while making some 350 revolutions per minute. The blow was received across the right shoulder, extending down below the scapula. At the time he felt no pain, and there was no soreness of the parts until about two weeks after, when he says he felt a lameness and soreness in the side, midway between the 7th and 8th ribs.

The soreness continued to increase for three months, when there appeared an abscess, which was opened by his attending physician. This abscess has continued to discharge ever since; sometimes the pus is thin, and sometimes of a thicker quality. At present, the patient complains of a pain in the right hypochondriac region. You notice, by percussion, that the hepatic dulness is about normal. If the liver had furnished this abscess, it would necessarily be enlarged; but, as it is about normal, I would say at once that the liver is not involved. The pain was not such as to indicate pleurisy; and, as the patient has had no cough, nor difficulty in respiration, we can safely say it is not pneumonia.

I should think, when the wheel struck him, it produced sufficient inflammation near the head of the rib to result in suppuration, the pus following along between the pleura and rib, until it could find a point of escape. It has never healed, owing to the denuded state of the rib, or portion of vertebra; most likely the former; nor will it heal until the necrosed or carious portion of the rib is removed, unless the pus changes its course.

The present symptoms are pain between the umbilicus and right hypochondriac region. There also seems to be an irregular contraction of the abdominal muscles. If you were to go directly down at the seat of pain, you would strike the upper part of the psoas muscle. There is already some swelling and tenderness in that part, increased by exercise.

The patient says that he feels so weak, and his back aches so badly towards night, that he can hardly sit up. Says he never had any cough, no headache to speak of. By careful introduction, the probe can be passed under the edge of the rib to its inner surface, and backward towards the junction of the rib with the spine. Patient says the abscess was opened three times, the last time about the first of September. The Doctor first introducing a director into the fistula, and cutting in the direction of the rib backwards. After which, he burned it out with caustic.

In this case, I have no hesitation in regarding its origin as at the junction of the rib with the vertebra. Up to the present time, the patient has kept a very good degree of health; but he says that he has lost flesh within the past two weeks, or since the pain commenced in the abdomen. From the existence of this abdominal pain, it is highly probable that the pus is taking a new direction down the spine, along the course of the psoas muscle. The spasmodic action of the abdominal muscles is evidently due to the irritation of the anterior branches of the lower intercostal nerves.

The disease is evidently caries (either of the head of the rib or of one of the vertebræ, probably the former); and the pus will be more likely to gravitate downward, ultimately appearing in the form of an abscess just below Poupart's ligament, as the symptoms are such as to plainly indicate present irritation and fulness in the upper part of the psoas region.

There are two methods of treatment that might be adopted in this case:—1. The surgical exsection of the rib, for the removal of the diseased bone. 2. The patient may be placed in a horizontal position, inclining a little towards the diseased side, and kept perfectly quiet for four or five weeks, with the internal administration of tonics and alterative remedies. You would accomplish two objects by this horizontal position, *viz.:* decrease the gravitation of pus downwards, and put the parts at rest. After this, I would recommend a spinal support, which will not admit of any rotary motion of the parts, and exactly the same as that employed in the treatment of any angular curvature; and then the patient may be allowed moderate outdoor exercise.

Laying surgical aid aside, I think this is the only treatment that will be apt to prove beneficial. Without treatment, he will continue to grow worse, and finally have an abscess at Poupart's ligament, becoming more feverish and emaciated daily.

As the attempt to exsect the head of the rib involves a serious operation, and we cannot be certain that the disease does not affect also the vertebræ, we should not advise an immediate resort to that method of treatment. By confining the patient to a strictly horizontal position, keeping the present opening as free as possible, and giving him three times a day a teaspoonful of the following tonic and alterative mixture, it is quite possible that the further extension of the suppurative process would be arrested, with a removal of all pain from the side and abdomen:—

℞. Tinct. Cinchonæ, ------------------ ℥ij.

Fl. Ext. Conium, ------------------ ℥j.

Bichlorid. Hydrarg., ---------------- 1 gr.

Mix.

After this prescription has been used three weeks, he may take in its place the syrup of iodide lime or iodide of iron.

Note.—*February 10th, 1869.*—The patient returned home the day following the above clinic, taking a letter to his physician. The treatment above indicated has been carried out to the present time, and information came from his attending physician two days since, saying that the patient was making good progress towards recovery.

